# Supplementary material for: Clinical Outcomes and Predictors of Mortality in Patients with Difficult-to-Treat Resistant Pseudomonas aeruginosa Infections: A Retrospective Cohort Study
Source: Antibiotics (Basel). 2026 Jan 1;15(1):33. doi: 10.3390/antibiotics15010033 (PMC12837929; doi:10.3390/antibiotics15010033)
Supplement: Supplementary file 1 [file antibiotics-15-00033-s001.zip › antibiotics-4048181-supplementary.pdf]

Figure S1. Model discrimination regarding clinical cure at end of therapy.

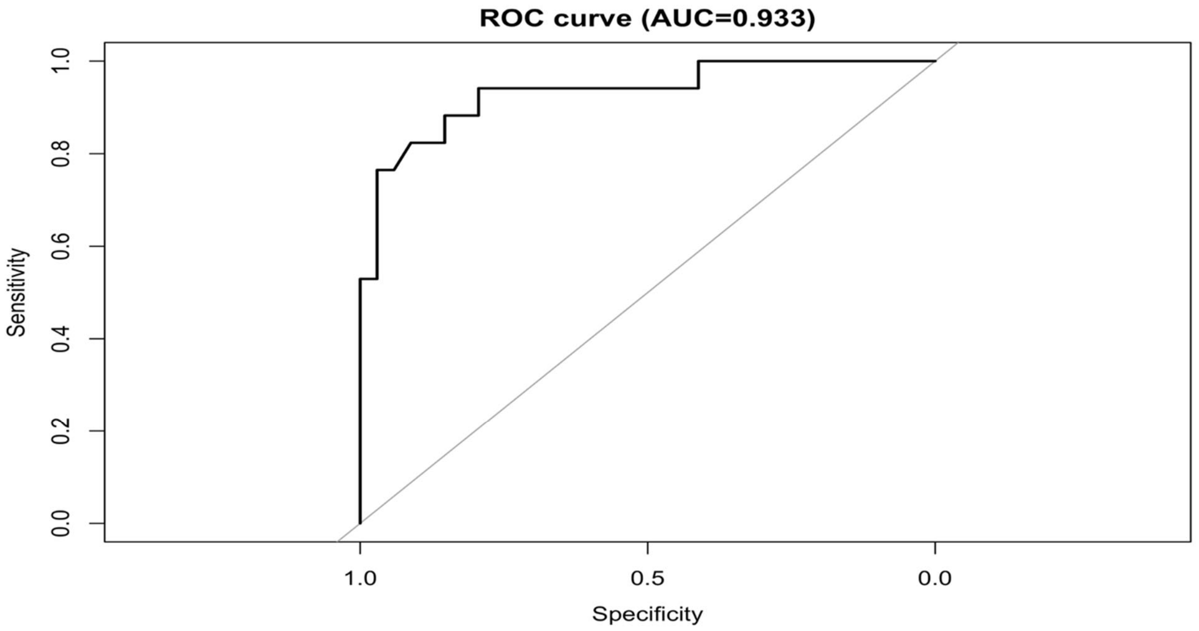

Figure S2. Model discrimination regarding microbiological cure at end of therapy.

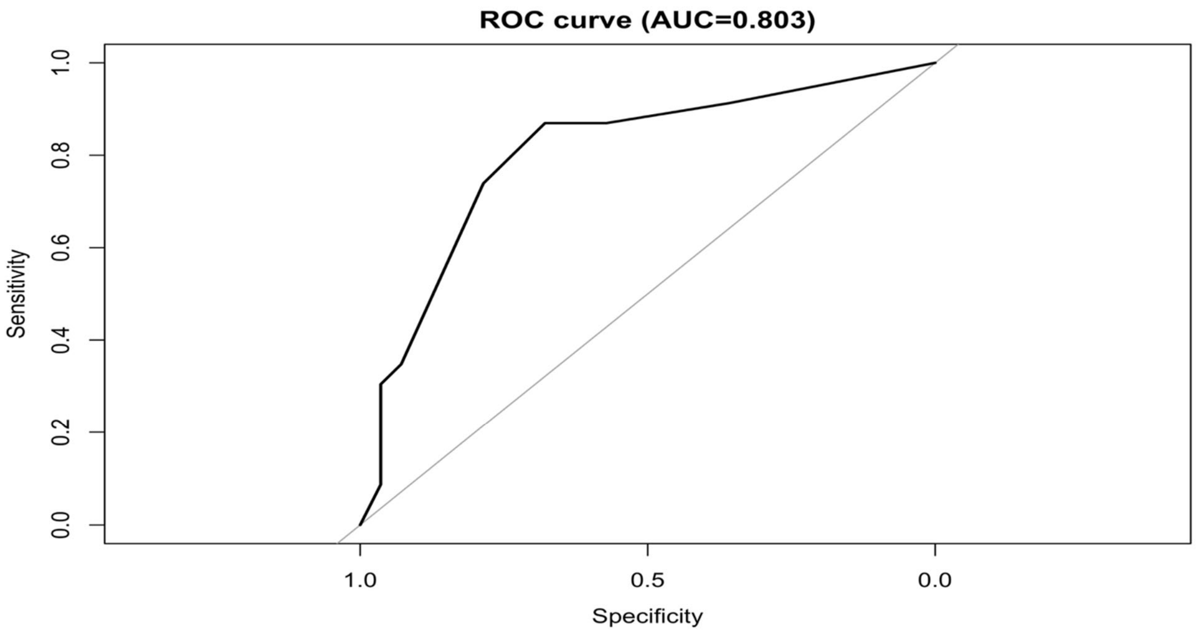

Figure S3. Model discrimination regarding 30-Day Infection-Related Mortality

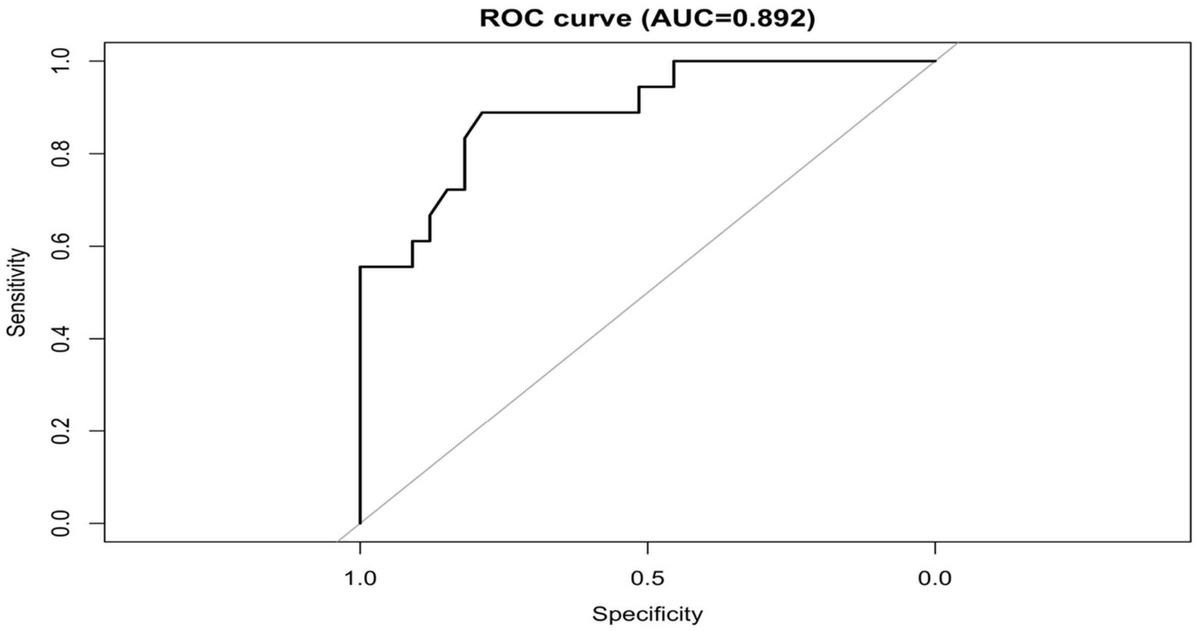

Table S1. Univariable and Multivariable Analyses for 30-Day Infection-Related Mortality

30-Day Infection-Related Mortality (Alive = 33, Died = 18)

**30-Day Infection-Related Mortality (Alive = 33, Died = 18)**					
**Variables**		**Univariable OR (CI 95%, p.value)**	**Multivariable aOR (CI 95%, p.value)**	**Final Model aOR (CI 95%, p.value)**	**Final Model with Firth aOR (CI 95%, p.value)**
Age		1.03 (0.99-1.07, p=.129)	1.15 (1.01-1.32, p=.041)	1.07 (1.01-1.17, p=0.059)	1.06 (1.00–1.12, p=0.063)
Ward of hospitalization at infection onset	Other				
	ICU	2.86 (0.69-11.92, p=.150)	0.08 (0.00-7.32, p=.272)		
Sex	Female				
	Male	1.30 (0.39-4.33, p=.669)			
Charlson Comorbidity Index		0.94 (0.79-1.11, p=.456)			
Previous hospitalization, last 3 months	No				
	Yes	0.43 (0.13-1.42, p=.168)	0.24 (0.02-2.82, p=.259)		
Previous antibiotic therapy, last 3 months	No				
	Yes	0.53 (0.16-1.71, p=.288)			
Immunodeficiency	No				
	Yes	0.28 (0.08-0.92, p=.036)	0.11 (0.01-1.94, p=.133)		
Previous Pseudomonas Infection, last 3 months	No				
	Yes	1.14 (0.34-3.83, p=.829)			

Previous surgery, last 3 months	No				
	Yes	5.89 (1.16-29.91, p=.032)	0.04 (0.00-34.57, p=.355)		
Mechanical Ventilation	No				
	Yes	2.34 (0.24-22.73, p=.462)			
Foley Urinary Catheter	No				
	Yes	2.34 (0.24-22.73, p=.462)			
Central Venous Catheter	No				
	Yes	1.70 (0.50-5.74, p=.395)			
Chest Drainage Tube	No				
	Yes	9087886.75 (0.00-Inf, p=.993)			
Septic Shock	No				
	Yes	9.33 (2.42-35.99, p=.001)	16.23 (0.50-531.20, p=.117)	10.4 (2.06-82.8, p=0.01)	7.02 (1.57–31.36, p=0.011)
Nosocomial Pneumonia	No				
	HAP	1.75 (0.13-23.70, p=.674)	13.37 (0.14-1315.46, p=.268)		
	VAP	5.83 (0.64-52.88, p=.117)	3769.26 (0.15-96292626.01, p=.112)		
Bloodstream Infection (BSI) or Catheter-Related Bloodstream Infection (CRBSI)	No				
	BSI	2.09 (0.50-8.76, p=.313)			
	CRBSI	0.84 (0.14-5.01, p=.845)			
Infectious Diseases Consultation	No				
	Yes	0.32 (0.05-2.14, p=.241)			

Adequate empirical therapy	Not empirical therapy				
	No	1.33 (0.29-6.14, p=.712)			
	Yes	1.40 (0.20-10.03, p=.738)			
Targeted therapy: Ceftazidime/Avibactam or Ceftolozane/Tazobactam vs Other	No targeted therapy				
	C/A or C/T	0.07 (0.01-0.36, p=.002)	0.00 (0.00-0.31, p=.019)	0.01 (0.00-0.16, p=0.003)	0.03 (0.00–0.30, p=0.003)
	Other	0.18 (0.04-0.87, p=.033)	0.01 (0.00-0.85, p=.042)	0.05 (0.00-0.39, p=0.011)	0.08 (0.01–0.58, p=0.013)
Sequential Organ Failure Assessment (SOFA) Score, Delta		1.25 (1.07-1.46, p=.005)	1.04 (0.75-1.43, p=.827)		
Polymicrobial Infection	No				
	Yes	1.43 (0.25-8.23, p=.690)			

Table S2. Models diagnostics for all the outcomes.

**Endpoint**	**AUC (95% CI)**	**R^2 Mc Fadden**	**Accuracy (95% CI)**
30-Day All-Cause Mortality	0.918 (0.846–0.990)	0.469	0.843 (0.714, 0.930)
30-Day Infection-Related Mortality	0.892 (0.801–0.985)	0.432	0.804 (0.669, 0.902)
Clinical cure at end of therapy	0.933 (0.857–1.000)	0.525	0.863 (0.737, 0.943)
Microbiological cure at end of therapy	0.803 (0.677–0.929)	0.213	0.765 (0.625, 0.872)
